# Effects of Diatomaceous Earth on House Dust Mite Sensitization in BALB/c Mice

**Published:** 2019-06-24

**Authors:** Sung-Yuan Liu, Yu-Hui Chang, Hui-Ru Ji, Cheng-Di Chiu

**Affiliations:** 1 Renai Biotechnology Limited, Taichung, Taiwan; 2 Green Polymer Corporation, Taichung, Taiwan; 3 Graduate Institute of Biomedical Science, China Medical University, Taichung, Taiwan; 4 School of Medicine, China Medical University, Taichung, Taiwan; 5 Department of Neurosurgery, China Medical University Hospital, Taichung, Taiwan

**Keywords:** Asthma, Diatomaceous earth, House dust mite, Allergy, Environmental exposure

## Abstract

**Background:**

House Dust Mite (HDM) is associated with hypersensitivity such as asthma. Patients with asthma benefit from improved living environment by reducing HDM exposure. In this study, we examined the effects of commercialized diatomaceous earth product, Casaggia® used as construction materials, on hypersensitivity in HDM-sensitized mice.

**Methods:**

Male mice were sensitized with house dust mite extract for 7d and then housed in diatomaceous earth (DE)-coated cages for 14 days at Animal Center of the Taichung Veterans General Hospital, Taichung, Taiwan in 2014. Levels of cytokine were determined using ELISA. White blood cell counts were recorded over 21d. Histological analysis was conducted to determine the remodeling of respiratory tract.

**Results:**

Exposure to DE resulted in a suppression in elevated eosinophilia induced by HDM in mice. In addition, elevated serum IgE responding to HDM sensitization were restored in the presence of DE. DE ameliorated the inflammation progression in airway.

**Conclusion:**

Environmental exposure to DE is suggested to benefit patients with hypersensitivity through relieving inflammatory symptoms. In a sense of prevention, DE represents a potential material against development of asthma.

## Introduction

Asthma known as respiratory allergic disease is characterized by excessive inflammatory response, structural remodeling, and obstruction in the airway. The prevalence of asthma has increased worldwide in past few decades, ranging from 1–18% (1, 2). Development of asthma is attributed to repeated exposure to aeroallergens including pollens and House Dust Mite (HDM) (3, 4). HDM is known as the most common cause of sensitization in asthmatic patients that approximately 85% of asthmatics are HDM allergic (5). HDM are found in dust and products with woven material or stuffing which provide suitable habitat for HDM. In addition, moisture that plays a vital role in natural habitation of HDM has been shown to contribute to development of allergic asthma. Despite the relevance of climatic factors including humidity to increase in asthma, the effects of manipulating climatic factor on HDM sensitization are still unclear.

Diatomaceous earth (DE) consists of predominately fossilized remains of diatoms which is a type of hard-shelled algae. It is commonly used for in a broad spectrum of purposes based on its chemical and structural properties such as filtration, abrasive and construction. DE has highly porous surface that makes it an ideal material for humidity control. Use of DE has been demonstrated to effectively reduce the growth of insects (6–8). In addition, as organic in origin, DE has been used to control internal parasites in livestock (9, 10). However, the effects of DE on moisture control associated with human disorder such as asthma is sketchy.

In the present study, we hypothesized that use of DE ameliorates HDM induced allergic responses. We examined physiological effects of DE at HDM-sensitized hypersensitivity in animal model. The changes of subtypes of blood cells and serum IgE levels after HDM challenge and pathological changes of lung tissue were analyzed.

## Materials and Methods

### Mice

To dispel the possibility that gender may influence allergic hypersensitivity, we chose male BALB/c mice aged 6 to 8-wk and weighing 20–25g as animal subjects to induce HDM sensitization. Mice were purchased from the BioLASCO Taiwan Co., Ltd and housed in environmentally controlled conditions (22 °C, a 12h light/dark cycle) with ad libitum access to standard laboratory chow and water. Prior to experiments, mice were habituated to new environment for one week. All experiments were performed at animal center of the Taichung Veterans General Hospital, Taichung, Taiwan in 2014.

The study protocol was reviewed and approved by the Research Ethics Committee of the Taichung Veterans General Hospital and all animal experiments were performed according to the institutional and state guidelines on the care and use of animals for experimental purposes.

Overall, 66 rats were used in the present study and allocated into 3 groups: mice without treatment (sham group), mice housed in a normal cage undergo HDM sensitization process (HDM group) and mice housed in a DE-coated cage undergo HDM sensitization process (HDM+DE group). Among them, 48 mice were assigned for histological examination and 18 mice were used for blood sampling at 4 different time points.

### Preparation of house dust mite crude extract

HDME was prepared by dissolving 1mg lyophilized HDM (Allergon AB, Angelholm, Sweden) in 1.0mL normal saline. Resulting solution was mixed with rotation for 90min and then centrifuged at 13000rpm for 15min. The supernatant was used as a crude extract of HDM (HDME). The protein concentrations of HDM were measured using a commercially available BCA protein assay kit (Pierce, USA) and adjusted to 100μg protein/mL for further use.

### Sensitization and aerosol challenge procedure

The mouse sensitization and aerosol challenge algorithm were performed (11). In brief, for mouse sensitization, 100μg/ml HDME was absorbed to 1mg/ml aluminum hydroxide [Al (OH)_3_] (Merck). The animals were administered intraperitoneally with 100μL HDM (100 μg/mL) and Al(OH)_3_ (1mg/mL) (n=6, the HDM group, and n=6, the DE group). After 7d post-administration, mice in the control, HDM+DE group and HDM group were housed separately in plastic cages of which plastic cages for DE group were coated with commercialized DE product (Casaggia®, GREEN POLYMER CORPORATION, Taichung, Taiwan). Animals were challenged by repeated exposures for 30min each day to an aerosol of HDM (100μg/mL) delivered at 0.5mL/min by a SUMO V-16 nebulizer (Japan) for 3d post-separation.

### White blood cell counts in mouse serum

Blood samples were obtained via the retro-orbital venous plexus on Day 1, 5, 7, and 14 post-exposure to HDM aerosol. Complete blood count was conducted using KX-21-Hematology Analyzer.

### ELISA

Total serum IgE was measured using a mouse IgE ELISA kit (BD Biosciences, USA) according to the instruction provided by the manufacturer. The total IgE concentration in each serum (ng/mL) sample was estimated using a standard sample from the manufacturer’s kit.

### Histological examination

The mice (n=4 in each group) were sacrificed on Day 1, 5, 7 and 14 post-exposure to HDM aerosol. Infusion was performed via the trachea with 4% paraformaldehyde. Lungs were excised and immersed in fresh fixative overnight. Lung tissue sections with a thickness of 2μm were obtained and stained with hematoxylin and eosin (H an E). Pulmonary lesions were categorized according to Shackelford et al. into five severity grades as following, 1=minimal (<1%), 2=slight (1–25%, +), 3= moderate (26–50%, ++), 4=moderate/severe (51–75%, +++), 5=severe/high (76–100%, ++++).

### Statistical analysis

Statistical comparisons were performed by analysis of variance (ANOVA) and turkey’s HSD post hoc tests. We used nonparametric statistics (Wilcoxon rank sum test) to test data differences between two groups if the data distribution is not corresponding Gaussian distribution (Kolmogorov-Smirnov test is significant). All values are shown as mean±standard deviation (SD). P< 0.05 was regarded as significant. All statistical analyses were performed using SPSS software (ver. 18.0, IBM Corp., Armonk, NY, USA).

## Results

### Effects of DE on differential WBC counts in HDM-sensitized mice

**Fig. 1.**
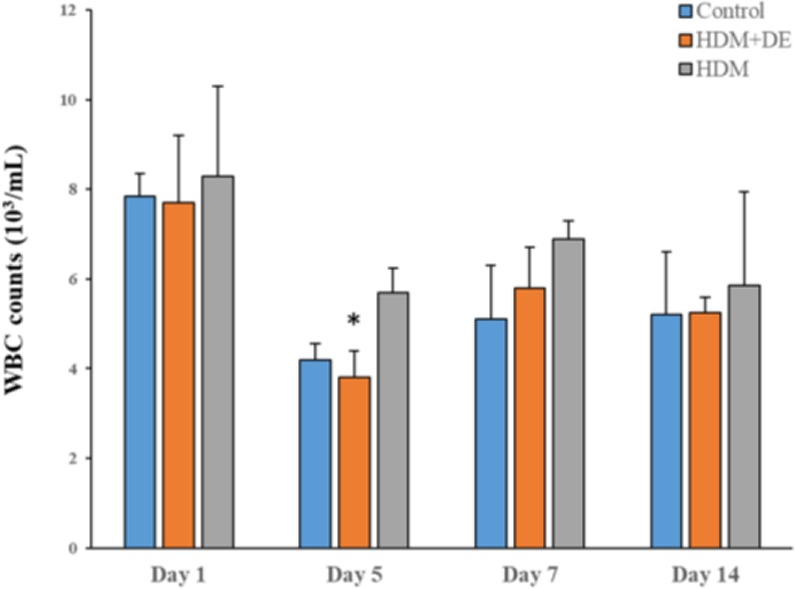
The effects of DE on peripheral total WBC counts. Blood samples were obtained from the HDM-sensitized mice on Day 1, 5, 7 and 14 post-sensitization (N=6). Data are presented as the mean ± SD. *P< 0.05

**Fig. 2.**
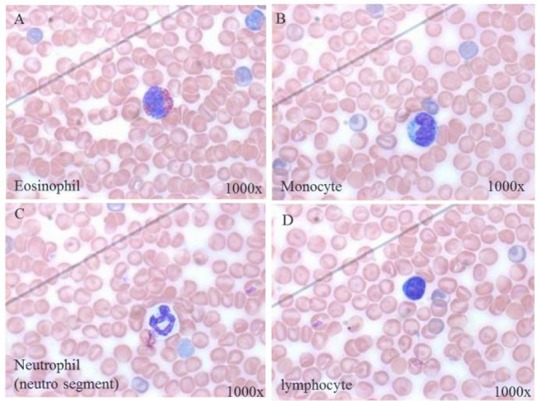
Subtypes of WBCs found in HDM-induced mice (1000×). It denotes (A) eosinophil, 0–4%, responsive for allergy, asthma, parasite; (B) monocyte, 3–8%, (C) neutrophil, 10–40%, (D) lymphocyte, 55–90%.

**Fig. 3.**
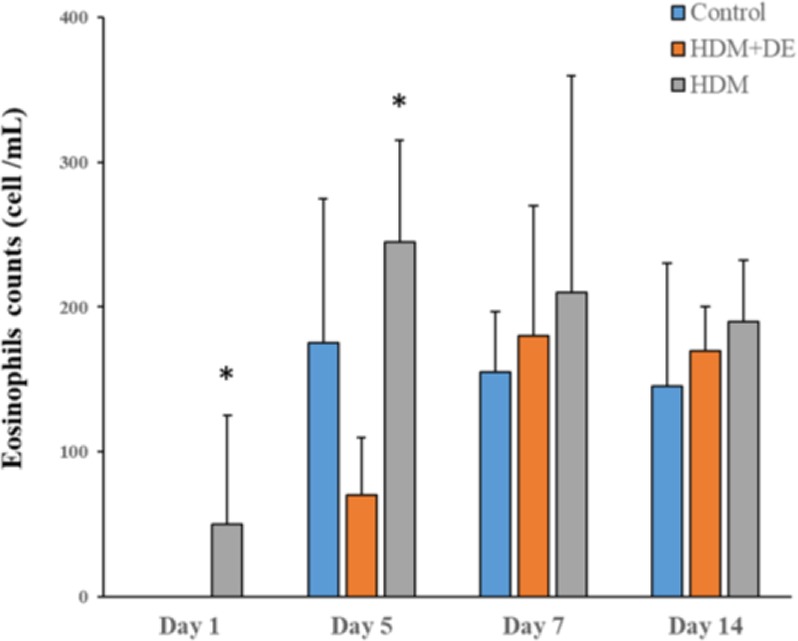
The effects of DE on peripheral eosinophil counts. Blood samples were obtained from the HDM-sensitized mice on Day 1, 5, 7 and 14 post-sensitization (N=6). Data are presented as the mean ± SD. *P< 0.05

To investigate whether DE abrogates HDM-induced eosinophils, peripheral total and differential WBC counts in HDM-sensitized mice were conducted. The peripheral total WBCs at 4-time points in 3 groups of mice with or without exposure to DE were comparable. A significant difference in total WBC counts was observed between HDM and DE groups on Day 5 post-exposure to HDM aerosol (P< 0.05) (Fig. 1). In addition, there were no significant differences in cell counts and morphological changes among neutrophil, monocyte, and lymphocyte (Fig. 2). Our data showed that eosinophil count in HDM+DE groups reached a peak on Day 7 post-sensitization, whereas both HDM and HDM+DE groups exhibited a peak on Day 5. The differential WBC counts revealed that HDM-sensitized mice exhibited a significantly higher number of eosinophils in the peripheral blood compared with those of HDM+DE groups at Day1 and Day 5 (P< 0.05) (Fig. 3).

### Effects of DE on IgE levels in HDM-sensitized mice

**Fig. 4.**
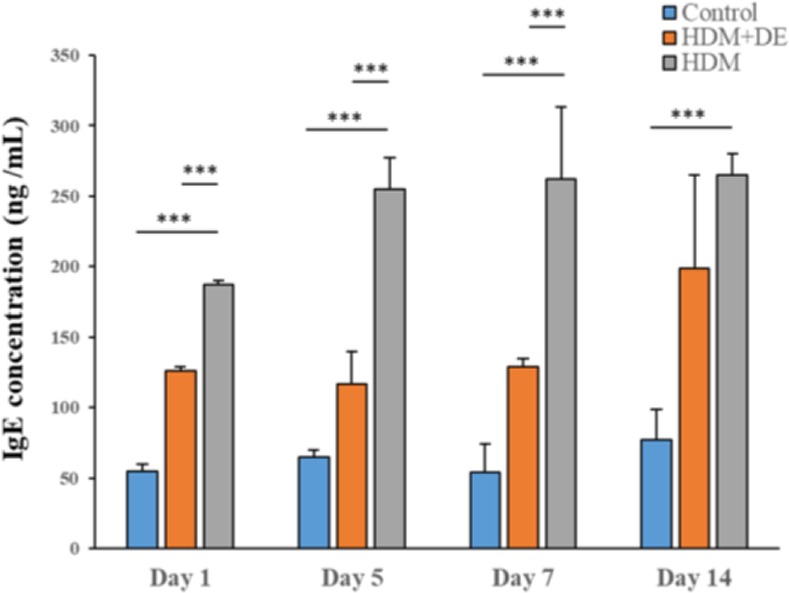
Blood IgE levels in mice after HDM sensitization. Blood samples were obtained from the HDM-sensitized mice on Day 1, 5, 7 and 14 post-sensitization. (ng/mL, n=6) Data are presented as the mean ± SD. ***P< 0.001

We next examined the effect of DE on HDM-induced increase in serum IgE levels. Exposure of mice to HDM led to markedly high serum IgE levels compared with those of the controls at 4 designated time points (P< 0.001) (Fig. 4). A significant difference in levels of serum IgE between HDM+DE and HDM groups on Day 1, 5 and 7 (P< 0.001). Interestingly, the levels of IgE in HDM+DE mice increased in a time-dependent manner.

### DE reduced HDM-induced lung inflammation

**Fig. 5.**
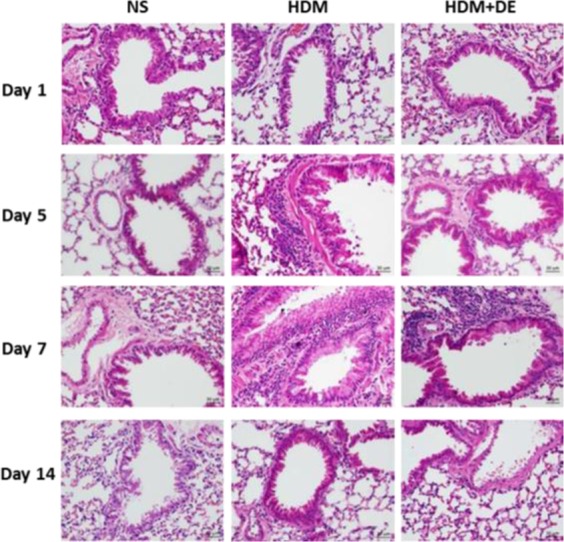
Pathological examination of inflammation in lung tissue among mice group of NS, HMD group and HMD+DE (H and E stain, 400×)

To investigate the mechanism by which DE was associated with decreased eosinophilia, we histologically assessed lung tissues of mice sensitized by HDM. HDM sensitization led to a slight inflammatory cell infiltration, mainly neutrophils and lymphocytes, fairly eosinophils and slight mucilage in the bronchial goblet cells, along with minimal macrophage infiltration in the alveolar space (Fig. 5). In HDM+DE mice, HDM-induced inflammatory cell infiltration and pathological appearance were observed on Day 7 post-sensitization with minor degree of severity compared with that of HDM mice (Table 1). Unexpectedly, the inflammatory response in lung tissue was absent in HDM-treated mice on Day 14 post sensitization.

**Table 1.** Summary of pathological incidence of lung after mite-induced mice

**Pathology/Degree^1^**	**Day/Groups (number, mice)**								

	**Day5**			**Day7**			**Day14**		

	**C**	**H+D**	**H**	**C**	**H+D**	**H**	**C**	**H+D**	**H**
**Inflammatory cell, perivascular and peribroncheal, focal (minimal to slight)**	-	-	2	-	2	2	-	-	-
**Infiltration, macrophage, alveolar, focal (minimal)**	-	-	1	-	1	1	-	-	-
**Mucification, goblet cell, bronchial, foal (slight to moderate)**	-	-	2	-	2	3	-	-	-

Degree of lesions was graded from one to five depending on severity: 1= minimal (< 1%), 2= slight (1–25%, +), 3= moderate (26–50%, ++)

## Discussion

We demonstrated that environmental exposure to DE leads to a reduction in degrees of HDM-induced hypersensitivity in vivo. Reduced eosinophilia was associated with the presence of DE. Moreover, the HDM-induced hypersensitive responses were ameliorated in the mice exposed to DE. Asthma is a prevalent chronic respiratory disorder in children and adolescents populations. HDM allergens are considered as important indoor allergens for humans inducing allergic diseases. Presence of HDM is suggested to be a risk factor for development of allergic asthma. HDM sensitization has been shown to contribute to the pathogenesis, progression, and severity of asthma. Use of HDM as model allergen for sensitization in animal models has been shown to be more ideal and logical than the other models such as ovalbumin (12). HDM-sensitized animal models display many features of allergic asthma including respiratory inflammation, airway remodeling and increased eosinophilia (13). We employed a HDM sensitization to establish an allergic airway inflammation model that exhibited typical features of allergic inflammation. HDM-associated increases in systemic eosinophilia were restored in presence of DE.

Systemic and local eosinophilic inflammation has been associated with severe asthma and poorer asthma control (14). Eosinophilia in blood is a good marker for asthma (15–17) and been used as a measurement of effects of anti-hypersensitive modalities. Airway eosinophilia is considered as a vital characteristic of early-onset allergic asthma, as well as the occurrence of late-onset non-allergic asthma (16). Our finding showed that DE reduced elevated systemic eosinophilia suggests that use of DE has the potential to improve the symptoms of allergic asthma. Unexpectedly, bronchial eosinophilia remained unaffected in the experimental setting. The finding is supported by the results of previous research, HDM-induced airway hypersensitiveness is not related to either eosinophil influx or allergen-specific serum IgE. In addition, a retrospective study has reported the absence of airway eosinophilia in patients with allergic asthma (18). However, further studies are required to determine the differences in the phenomenon of eosinophil counts in HDM-sensitization modal.

Increasing evidence has highlighted the potential use of DE for insect control (19–21). Recent studies have demonstrated the insecticidal effect of DE against different species of mites in animal (10, 22). The insecticidal effects of DE have been postulated to be attributed to rapid and lethal dehydration of insect. We explored the use of Casaggia®, a new DE formulation, for improving the hypersensitive response induced by HDM. Animals of HDM+ DE group exhibited relatively lower eosinophil counts and levels of total IgE in blood compared with those of mice sensitized with HDM, suggesting that asthmatics can benefit from avoidance of environmental risk factor using DE. However, in this study, animals were sensitized with HDM extract and housed in DE-coated cages without direct exposure to dust mites. Moreover, increased eosinophilia in HDM-sensitized rats declined slightly over time. A possible explanation for the anti-hypersensitive effect in presence of DE is that use of DE leads to a moisture loss of surrounding environment, which inhibits the growth of microorganisms acting as challenge to HDM-sensitized animals. Nevertheless, further studies are necessary to elucidate the mechanism underlying the effect of DE on hypersensitivity. Long-term occupational exposure of workers in the crystalline silica was found to have an increased risk of silicosis (23). Despite current commercial, DE product contains mostly amorphous silica but no crystalline silica, there still are doubts for long term usage. Further investigation for the effect of commercial DE products to human lung is necessary.

## Conclusion

Exposure to DE ameliorates elevated eosinophilia and total IgE in animals sensitized with HDM. DE represents a potential approach to prevent occurrence of hypersensitivity and to improve the symptoms of allergic asthma.
